# The Micro-Structure of the Celiac Ganglia—A Two-Photon Microscopy Study on Parkinson’s Disease

**DOI:** 10.3390/diagnostics15060659

**Published:** 2025-03-08

**Authors:** Diana-Theodora Morgos, Lucian-George Eftimie, Horia Nicolae, Remus Iulian Nica, Constantin Stefani, Daniela Miricescu, Radu Hristu, George A. Stanciu, Adrian Tulin, Florin Filipoiu

**Affiliations:** 1 Department of Anatomy, Doctoral School, Carol Davila University of Medicine and Pharmacy, 050474 Bucharest, Romania; theodora.morgos@drd.umfcd.ro; 2 Department of Anatomy and Biomechanics, National University of Physical Education and Sports, 060057 Bucharest, Romania; 3 Department of Neurology, Carol Davila University of Medicine and Pharmacy, 050474 Bucharest, Romania; horia.nicolae@umfcd.ro; 4 Department of Neurology, Elias University Emergency Hospital, 011461 Bucharest, Romania; 5 Discipline of General Surgery, Faculty of Midwifery and Nursing, Carol Davila University of Medicine and Pharmacy, 050474 Bucharest, Romania; remus.nica@umfcd.ro; 6 Surgery Department, Central Military Emergency University Hospital “Dr. Carol Davila”, 010825 Bucharest, Romania; 7 Department I of Family Medicine and Clinical Base, Central Military Emergency University Hospital “Dr. Carol Davila”, 010825 Bucharest, Romania; constantin.stefani@umfcd.ro; 8 Discipline of Biochemistry, Faculty of Dentistry, Carol Davila University of Medicine and Pharmacy, 8 Eroii Sanitari Blvd, 050474 Bucharest, Romania; 9 Center for Microscopy-Microanalysis and Information Processing, National University of Science and Technology Politehnica Bucharest, 060042 Bucharest, Romania; radu.hristu@upb.ro (R.H.); stanciu@physics.pub.ro (G.A.S.); 10 Discipline of Anatomy, Carol Davila University of Medicine and Pharmacy, 8 Eroii Sanitari Blvd, 050474 Bucharest, Romania; adrian.tulin@umfcd.ro (A.T.); florin.filipoiu@umfcd.ro (F.F.)

**Keywords:** two-photon microscopy, celiac ganglia, neurodegeneration, Parkinson’s disease

## Abstract

**Background/Objectives**: This study explores the micro-structure of celiac ganglia using two-photon microscopy (TPM) to highlight histological features in neurodegenerative conditions. Neurodegenerative diseases like Parkinson’s disease (PD) are linked to dysautonomia, impacting autonomic regulation and leading to significant gastrointestinal and autonomic symptoms. Our research compares imaging results from TPM and SHG microscopy, visualizing neuronal integrity, collagen distribution, and the architectural organization of celiac ganglia. SHG specifically allows detailed imaging of collagen fibers and neuronal structures, revealing alterations in collagen density and organization that correlate with dysautonomia. **Methods**: The cross-sectional study was conducted at “Dr. Carol Davila” Central Military Emergency University Hospital, Bucharest, Romania, involving 70 participants diagnosed with PD (Hoehn and Yahr stages 2–4), including 35 with dysautonomia and 35 without. We utilized samples from PD patients with and without dysautonomia, applying immunohistochemical markers for sympathetic neurons. **Results**: Our findings reveal significant pathological changes in neuronal structure and collagen architecture. Immunohistochemical markers (neuropeptide Y, neurofilament heavy chain (NF-H), and tyrosine hydroxylase) were employed to characterize sympathetic neurons, while TPM and SHG provided high-resolution imaging of neuronal integrity and extracellular matrix composition. **Conclusions**: These imaging techniques present a promising tool for early diagnosis and assessment of neurodegeneration and dysautonomia in PD patients. Moreover, these techniques may represent a critical bridge between histopathological findings and clinical manifestations, underscoring their role in enhancing our understanding of neurodegeneration and autonomic dysfunction in Parkinson’s disease.

## 1. Introduction

In the field of clinical neurology, there is a pressing need for non-invasive and faster diagnosis methods for neurodegeneration. Dysautonomia is a form of sympathetic and parasympathetic ganglia neurodegeneration, and it is characterized by an imbalance between the two components of autonomic nervous system. The celiac ganglia, a major component of the sympathetic nervous system, play a critical role in autonomic regulation of gastrointestinal functions [1].

Neurodegenerative diseases, like Parkinson’s disease (PD), often exhibit dysautonomia, where dysfunction in the celiac ganglia contributes to gastrointestinal symptoms and overall autonomic dysregulation. Symptoms of dysautonomia encompass postural orthostatic tachycardia and orthostatic hypotension, which is the most distinctive feature of the condition. Other symptoms may include dizziness or lightheadedness, abnormal sweating, gastrointestinal issues such as constipation or diarrhea, urinary retention, sensations of warmth, and nausea [2].

PD is a neurodegenerative disorder characterized by the degeneration of dopaminergic neurons in the substantia nigra, leading to motor symptoms such as tremors, rigidity, and bradykinesia [3]. Dysautonomia often occurs in PD patients. This condition can manifest as orthostatic hypotension, gastrointestinal disturbances, and bladder dysfunction, which significantly impact quality of life. The interplay between PD and dysautonomia involves both direct neurodegeneration and the accumulation of alpha-synuclein aggregates, which can affect autonomic centers in the brainstem [4].

Emerging research suggests that degeneration of neurons in the celiac ganglia may exacerbate neurodegenerative processes. This condition represents a form of autonomic failure and is clinically observed as a result of impairment to neuronal populations. It is associated with decreased quality of life for affected individuals and contributes to significant social burden [5].

One approach in dysautonomia research is nonlinear optical (NLO) microscopy techniques, which uses nonlinear light–matter interactions to generate high-contrast images of tissues without the need for labeling. NLO microscopy techniques that involve the simultaneous interaction of two photons with the sample are classified as two-photon microscopy methods (TPM). Two prominent TPM techniques are two-photon excited fluorescence microscopy (TPEF) and second harmonic generation microscopy (SHG). TPEF involves the simultaneous absorption of two photons, leading to the emission of a single photon with a slightly smaller energy. SHG, on the other hand, generates a new photon with twice the energy of the initial photons through the interaction of two photons with a nonlinear material [6].

SHG is a microscopy technique that utilizes the optical properties of nonlinear interactions to obtain high-resolution images of fibrillar collagen in various tissues. It is particularly useful for imaging structures with a non-centrosymmetric arrangement—such as collagen fibers—making it invaluable for biological studies and for the assessment of structural integrity and potential associated pathologies [7].

TPEF is a technique that usually uses near-infrared light to excite fluorescent molecules in a sample. This allows for high-resolution imaging with minimal photobleaching and photodamage and increased depth penetration. It can provide high-resolution images of cells and their surrounding environment, while SHG can image fibrillar structures like collagen [8].

Differences between TPEF and SHG microscopy include the excitation mechanism—TPEF uses simultaneous two-photon absorption, while SHG microscopy uses nonlinear optical interactions, TPEF requires labeling with fluorescent dyes or proteins but can also image endogenous fluorescence, while SHG microscopy does not require labeling [9,10]. TPEF is commonly used for imaging labeled samples in neuroscience, cell biology [11], and developmental biology, while SHG microscopy is often used for imaging collagen and other biological structures in tissue engineering, cancer research, and connective tissue biology [12].

To investigate the micro-structure of the celiac ganglia, we employed TPEF and SHG imaging microscopy techniques. SHG microscopy serves as a powerful tool for examining the intricate microarchitecture of celiac ganglia, located near the abdominal aorta. These nerve clusters are critical parts of the autonomic nervous system, involved in the innervation of abdominal organs. SHG allows researchers to investigate the structural characteristics of celiac ganglia, including nerve fiber organization, collagen distribution, and interactions with surrounding tissues [13]. TPEF microscopy provides high-contrast and high-resolution images of metabolic substrates and structural proteins without the need for exogenous fluorescent labels [14].

The combination of TPEF and SHG microscopy enables researchers to gain insights into the micro-structure of celiac ganglia at multiple scales, from the organization of nerve fibers to the distribution of collagen and other extracellular matrix components [15].

Immunohistochemistry (IHC) and TPM are complementary techniques utilized in biological research and diagnostics, each providing insights into tissue architecture and cellular characteristics. IHC involves using specific antibodies to detect the presence and localization of proteins within tissue sections, allowing for visualization of cellular components and identification of specific cell types based on their protein expression profiles. This technique is crucial for understanding the molecular composition of tissues, including markers indicative of neuronal integrity, such as neurofilament heavy chain (NF-H), neuropeptide Y (NPY), and tyrosine hydroxylase (TH) in sympathetic neurons.

In this study, we propose an application for TPEF and SHG imaging of sympathetic ganglia in order to assess early alterations in autonomic ganglia morphology in PD patients with and without dysautonomia. Combining IHC for neuronal markers with TPM elucidates protein expression and tissue integrity, pivotal in dysautonomia.

Our aim is to address the early detection of pathological changes in sympathetic ganglia associated with PD by utilizing the complementary strengths of TPEF and SHG microscopy. By analyzing the morphological data obtained through these imaging techniques, we aim to identify specific alterations in collagen architecture, neuronal structure, and the extracellular matrix that may precede clinical symptoms of dysautonomia. This integrative approach is intended to enhance our understanding of the disease’s progression, potentially leading to improved management strategies for patients at risk of neurodegenerative disorders. Ultimately, our research seeks to bridge the gap between histopathological findings and clinical manifestations.

Our study hypothesizes that early alterations in the morphology of sympathetic ganglia can be detected through advanced imaging techniques, particularly TPEF and SHG microscopy, which have the potential to reveal subtle but significant changes in collagen organization, neuronal integrity, and extracellular matrix composition. We plan to compare sympathetic ganglia from PD patients exhibiting dysautonomia against those without, focusing on quantifying features such as collagen fiber density and orientation, neuronal cell body size, and dendritic morphology.

## 2. Materials and Methods

In this study, we used TPEF and SHG microscopy to image a neurodegenerative condition characterized by dysfunction of the autonomic nervous system—dysautonomia in PD.

### 2.1. Study Design

This study employs a cross-sectional design aimed at exploring the morphological alterations within sympathetic ganglia associated with dysautonomia in PD. By utilizing advanced imaging techniques—TPM—the study aims to correlate early pathological changes with clinical manifestations of dysautonomia.

The study was conducted at “Dr. Carol Davila” Central Military Emergency University Hospital, Bucharest, Romania. The timeline included recruitment of patients from May 2024 to October 2024. Data collection commenced after obtaining ethical approval and informed consent, with the exposure period being part of routine clinical assessments performed at the hospital. Follow-up data were gathered through clinical evaluations at prescribed intervals during this period.

### 2.2. Participants

Eligibility Criteria: Participants included individuals diagnosed with PD, specifically in Hoehn and Yahr stages 2–4, aged 45–80 years. Inclusion criteria are confirmed diagnosis of PD and presence of dysautonomia based on specified clinical diagnostic criteria. The impairment of balance is the most frequent clinical sign of dysautonomia in PD. Clinical diagnostic of dysautonomia include significant orthostatic hypotension, a decline in blood pressure of 20/10 mmHg or greater within three minutes of standing upright with a heart rate that does not increase or drops significantly within three minutes of standing upright and autonomic symptoms: excessive sweating, warmth, nausea.

PD is quantified within Hoehn and Yahr stages, reflecting the severity of symptoms. Stage 2 means that symptoms are bilateral without impairment of balance, stage 3 means bilateral disease with impairment of balance, stage 4 reveals a severe disease with disabling impairment of balance. Exclusion criteria are presence of other significant comorbidities affecting autonomic function, neurological disorders other than PD, recent surgical interventions affecting sympathetic nervous system function and patients who declined participation or could not provide informed consent.

Sources and Methods of Selection: Participants were recruited from outpatient clinics within the hospital, in neurology department, ensuring a diverse patient population reflective of the regional demographic.

### 2.3. Variables

Outcomes are pathological changes in collagen architecture, neuronal structure, and extracellular matrix in sympathetic ganglia, assessed through TPM. Exposures are diagnosis of dysautonomia in individuals with PD, evaluated through clinical symptoms and orthostatic testing results.

Predictors are clinical indicators such as blood pressure changes during orthostatic tests and reported autonomic symptoms. Potential confounders are age, duration of PD, and medication type and dosage, and comorbidities were considered as potential confounding variables. Effect modifiers are severity of dysautonomia symptoms and stage of PD were assessed as potential modifiers of the outcomes.

### 2.4. Data Sources and Measurement

Data gathering consisted of clinical evaluations including orthostatic test results to confirm the presence of dysautonomia, histological examination of tissue samples (celiac ganglia) using established IHC markers (NF-H, NPY, and TH), and imaging data sourced from bright-field TPM.

Measurement of variables implied the following: the presence and quantitative evaluation of histological markers were measured through standardized protocols in histopathology, and image analysis was conducted using software developed for image alignments and quantitative comparisons.

Comparability of assessment methods means that each group (PD with dysautonomia vs. without) was assessed using the identical processing protocols to ensure homogenous measurement across groups.

### 2.5. Bias

Efforts to address potential sources of bias included blinding of pathologists to the clinical status of patients during the histological and imaging analysis to minimize observer bias, standardization of imaging techniques and protocols to maintain consistency in data collection, and performing statistical adjustments for recognized confounders in the analysis phase to account for variations in age, duration of disease, treatments, and other variables.

### 2.6. Study Size

The study size was 70 participants (35 per group—PD with dysautonomia and PD without dysautonomia).

### 2.7. Ethics:

The Ethics Committee at Carol Davila University Central Emergency Military Hospital in Bucharest, Romania, approved the use of patient samples for research purposes (approval no. 591/24 May 2023), and written informed consent was secured from all subjects. All procedures were conducted in accordance with relevant guidelines and regulations, in alignment with the Declaration of Helsinki.

### 2.8. Sample Processing Protocol

The study protocol involved a baseline evaluation consisting of physical examination of the patients in order to confirm or infirm dysautonomia, and subjects underwent orthostatic test to assess the above-mentioned criteria. We collected, with echo guided fine needle aspiration, tissue samples of celiac ganglia from patients with PD and dysautonomia and PD without dysautonomia, and we compared them through IHC and TPM.

Thin serial sections (4–7 μm) were prepared from formalin-fixed, paraffin-embedded tissue blocks, mounted on glass slides, and stained either with hematoxylin and eosin (H&E) or according to established IHC protocols. Tissue fixation is essential for preserving the structural integrity of biological samples, achieved by immersing fragments in 10% formalin for one hour during the initial phase and two hours in a subsequent phase. Following fixation, dehydration is conducted to eliminate residual fixative and cellular water through a series of alcohol baths with increasing concentrations (70%, 80%, 96%, and 100%), with each stage lasting one hour. The next step, clearing, involves removing alcohol and dissolving lipids using a paraffin solvent such as toluene over three separate baths, each lasting one hour. The process culminates with paraffin inclusion, which entails impregnating tissues with paraffin wax to form a block, thus, facilitating thin sectioning during microtomy. Microtomy involves slicing the paraffin-embedded blocks into thin sections measuring 2–4 μm, which are then floated on a warm water bath to eliminate wrinkles before being mounted on glass slides. The deparaffination stage follows, which removes wax by heating the sections for 20–30 min, with an additional step in heated toluene for the same duration. The hydration process then prepares the tissue for staining by transitioning through decreasing concentrations of alcohol (100%, 96%, 80%, 70%) over five minutes each, finally ending in water. H&E staining is performed, wherein nuclei are stained blue with hematoxylin for five minutes, followed by differentiation in tap water and subsequent eosin staining for another five minutes. The final steps involve dehydration and clearing before mounting the slides with a low viscosity xylene-based medium and applying glass coverslips for preservation. Additional tissue samples underwent the same processing steps as the H&E samples, excluding the staining steps [11]. Trained pathologists identified regions of interest (ROIs) for TPM imaging.

To aid the differential diagnosis of dysautonomia, we utilized specific IHC markers. Sympathetic neurons from celiac ganglion showed positivity for NF-H, which is characteristically expressed in peripheral sympathetic neurons, as it contains neurofilament proteins. We also included IHC staining for NPY. A commonly used IHC marker for sympathetic neurons is also TH. This enzyme is critical in the synthesis of catecholamines (dopamine, norepinephrine, and epinephrine) and is typically expressed in sympathetic neurons.

Slides were analyzed using a Zeiss Axio Imager M2 bright-field microscope (Carl Zeiss Microscopy GmbH, Germany, Munchen) to identify regions of interest (ROIs), which were subsequently imaged using TPM.

For combined imaging of SHG and TPEF, we utilized a Leica TCS-SP confocal laser scanning microscope adapted for NLO imaging. The excitation source was a Ti–Sapphire laser (Chameleon Ultra II, Coherent, Dierburg, Germany) set to 870 nm, emitting ~140 fs pulses at a repetition rate of 80 MHz. The laser beam was circularly polarized by an achromatic quarter-wave plate (AQWP05M-980, Thorlabs, Newton, NJ, USA) and an achromatic half-wave plate (AHWP05M-980, Thorlabs) positioned in the laser path before entering the microscope. A 40× magnification objective with a numerical aperture (NA) of 0.75 was employed to focus the laser beam on the sample and to collect TPEF signals, while SHG signals were captured in the forward direction using a condenser lens with a NA of 0.9. The SHG signals were filtered using a shortpass filter (FF01-750/SP-25, Semrock, Rochester, NY, USA) in conjunction with a bandpass filter (FB430-10, Thorlabs) placed before the detector. The resulting images are displayed as composite images, with TPEF signals shown in magenta and SHG signals in green; areas of co-localized TPEF and SHG signals appear in white.

Bright-field microscopy images were acquired on H&E and IHC-stained sections using three distinct markers. Different ROIs identified on H&E-stained sections were further imaged using TPM on unstained tissue sections. Although these stained and unstained sections were sourced from the same histological block and comprised identical tissue structures, minor morphological variations occurred across sections. TPM image tiles were acquired and stitched together to create a mosaic covering a larger tissue area.

### 2.9. Statistics

Statistical analysis was performed using SPSS 26.0 for Windows and Microsoft Excel 2019. Quantitative variables were tested for normal distribution using the Shapiro–Wilk Test and were written as averages with standard deviations or medians with interquartile ranges. Differences between groups of quantitative variables with non-parametric distribution were tested using the Mann–Whitney U test. The threshold considered for the significance level for all tests was considered to be α = 0.05.

## 3. Results

### 3.1. Statistical Results

Demographic characteristics and Hoehn and Yahr stages distribution related to dysautonomia are shown in the table below (Table 1).

Hoehn and Yahr stages distribution are shown in the table below (Table 2).

The descriptive statistics for the Hoehn Yahr scale indicate the number of cases examined across three stages of PD. For Hoehn Yahr stage 2, there are 20 cases with a mean score of 10.00 and a standard deviation of 1.414. In stage 3, there are 31 cases with a higher mean of 15.50 and a greater standard deviation of 2.121. For stage 4, there are 19 cases with a mean of 9.50 and a lower standard deviation of 0.707. The valid *N* (total) of 70 indicates a robust sample size.

Furthermore, we analyzed with Bayesian Statistics the value of the specific outcome for Hoehn Yahr 2 with dysautonomia and Hoehn Yahr 3 with dysautonomia, which are the most frequent stages of PD (Table 3).

The Bayesian estimates of group means provide a probabilistic understanding of the Hoehn Yahr scale across different stages of PD. The posterior distribution of each group’s mean reveals that Hoehn and Yahr 2 with dysautonomia has a central tendency around 10.05 with 95% believable range (credible interval) from 9.48 to 10.63. Similarly, Hoehn and Yahr 3 with dysautonomia has a mean of 15.42 with a corresponding credible interval of 14.85 to 15.99. This Bayesian approach indicate a clear distinction between the average Hoehn and Yahr scores of 2 and 3 stages, with the latter stage showing significantly higher scores, which is consistent with the clinical understanding of PD progression. The analysis of the Hoehn Yahr scale presents an overview of the data corresponding to stages 2 and 3 of PD without dysautonomia. For Hoehn Yahr 2, the mean score is 10.05 with a standard deviation of 4.353. In contrast, Hoehn Yahr 3 shows a higher mean score of 15.42 and a larger standard deviation of 6.529.

There are similar results, statistically, between the PD with dysautonomia and PD without dysautonomia. Moreover, we used inferential statistics to draw a conclusion about a population based on sample data (Table 4).

For Hoehn Yahr stage 2, the mode is 5.25, and the mean is slightly higher at 5.50, with a variance of 1.375. The 95% credible interval ranges from 3.45 to 8.03. Hoehn Yahr stage 3 exhibits a higher mode of 8.00 and a mean of 8.25, accompanied by a greater variance of 2.063. The credible interval for this stage spans from 5.68 to 11.29. Meanwhile, Hoehn Yahr stage 4 has a mode of 5.00 and a mean of 5.25, with a variance of 1.313, and a credible interval from 3.25 to 7.72. The use of a Gamma (2, 2) prior for the Poisson rate/intensity reinforces the Bayesian framework employed to model these rates, allowing for a coherent interpretation of the estimated distributions derived from the data.

This distribution of Hoehn Yahr stages suggests a statistical equilibrium, where the observed variations in PD symptom severity are balanced among the groups. The mean and standard deviation of Hoehn Yahr stage 2 and stage 4 are more homogeneous, indicating a tighter distribution of scores and minimal deviation from the mean. Stage 3 exhibits a higher degree of heterogeneity, suggesting a more heterogeneous sample. This equilibrium among the groups is further reinforced by the robust sample size (*N* = 70), which enhances the reliability and generalizability of these findings, providing insights into the underlying patterns of PD symptom severity across the stages. Quantitative analysis based on the imaging data, when comparing autonomic dysfunction with controls, was conducted (Table 5).

**Figure 1:**
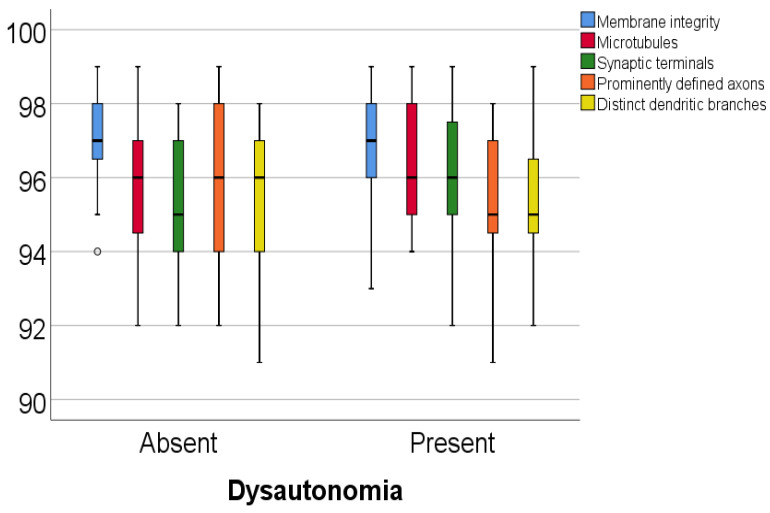
Comparison of membrane integrity, microtubules, synaptic terminals, prominently defined axons, and distinct dendritic branches percentages according to the existence of dysautonomia.

**Figure 2:**
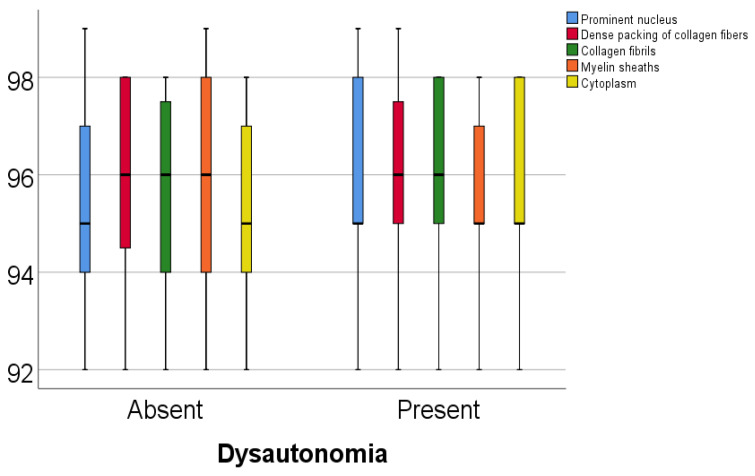
Comparison of prominent nucleus, dense packing of collagen fibers, collagen fibrils, myelin sheaths, and cytoplasm percentages according to the existence of dysautonomia.

Data from Table 5 and Figure 1 and Figure 2 show the comparison of analyzed parameters according to the existence of dysautonomia. Distribution of the analyzed parameters was non-parametric in one or both groups according to the Shapiro–Wilk test (*p* < 0.05). According to the Mann–Whitney U tests, all differences observed were not statistically significant (*p* > 0.05), as such, in patients with Parkinson’s disease, according to these data, existence of dysautonomia was not significantly associated with any abnormal modification of the celullar elements analyzed in the study.

### 3.2. Microscopy Results

**Figure 3:**
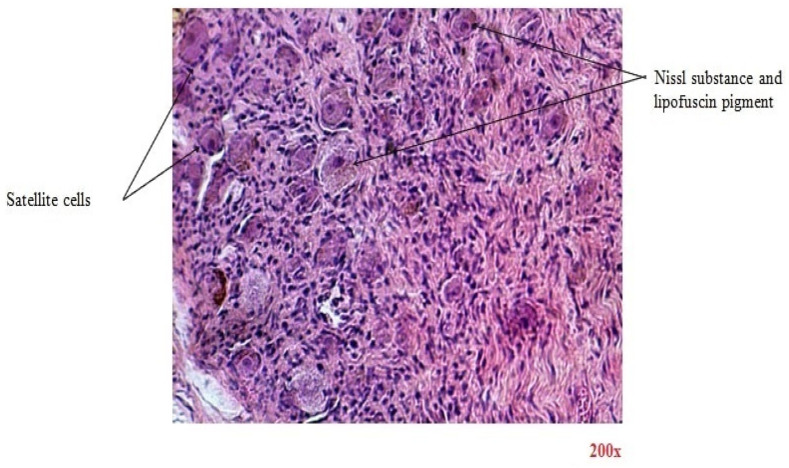
Transverse section through celiac ganglion (H&E-stained section, magnification 200×).

There are similar results in terms of microscopic imaging between the two categories. In Figure 3, the H&E-stained section reveals the ganglion that is encased in fibrous tissue sheaths that surround the components of the nervous structures. Ganglion cells are large nerve cell bodies with large excentric nuclei and prominent nucleoli. Their cytoplasm contains abundant basophilic Nissl substance, which represents the granular endoplasmic reticulum and ribosomes. Lipofuscin yellow-brown pigment granules can also be found in some nerve cell bodies. At the periphery of nerve cell bodies, small round satellite cells (glial cells) are found. Nerve fibers are defined by non-myelinated and myelinated axons of different diameters seen in the cross-section. Schwann cells are glial cells responsible for insulating axons in the peripheral nervous system by forming the myelin sheath. This sheath, which appears pink, encases myelinated axons, facilitating efficient nerve signal transmission. Interspersed within the nervous tissue are fibroblasts, which contribute to structural support and provide a context for the spatial organization of nerve cells.

**Figure 4:**
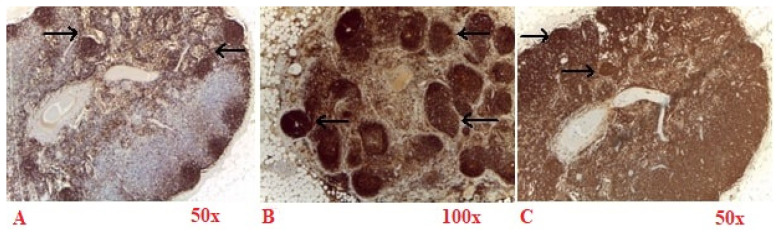
Immunohistochemical staining for neuropeptide Y (NPY) (magnification 50×) (**A**), neurofilament heavy chain (NF-H) (magnification 100×) (**B**) and tyrosine hydroxylase (TH) (magnification 50×) (**C**). In (**A**), Schwann cells are marked with arrows; In (**B**,**C**), arrows indicate sympathetic neurons.

**Figure 5:**
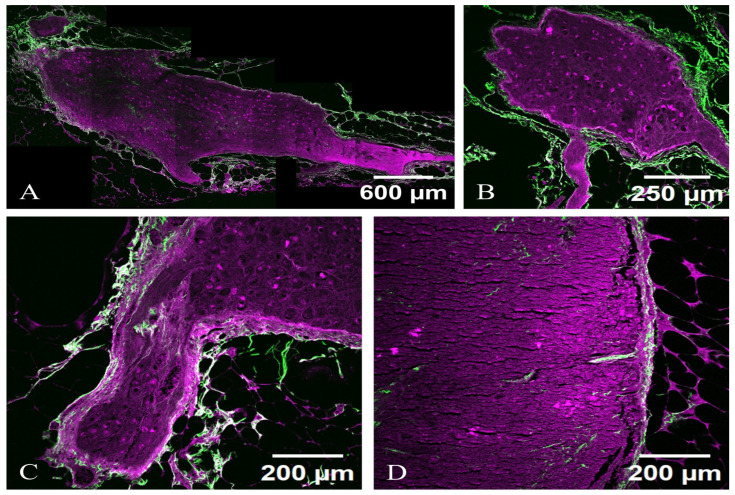
TPM images of sympathetic neurons from celiac ganglion. (**A**,**B**) Imaged area closer to the axon. (**C**) Imaged area closer to the dendrites. (**D**) Sympathetic neurons appear with stronger fluorescence compared with Schwann cells and fibroblasts. TPEF signals are shown in magenta—meaning dense cytoplasm with microtubules, while SHG signals are displayed in green—meaning dense packing of collagen fibers.

Sympathetic neurons exhibit strong membrane labeling when stained with an antibody against NPY (Figure 4A), NF-H (Figure 4B) and complete membrane labeling TH (Figure 4C), and a marker specifically expressed in sympathetic neurons and in dopaminergic neurons. These labeling are shown in walnut or brown in the bright-field microscopy images in Figure 4. These cells can also be effectively highlighted in TPM images, appearing with magenta (TPEF) and green (SHG) in Figure 5B.

Sympathetic neurons display intense positivity for NPY (Figure 4A) and are clearly visible in the TPM images of NPY stained tissue sections located near the axon (Figure 5A). In contrast to NF-H (Figure 4B) stained sections, where sympathetic neurons manifest pronounced membrane brightness, the differentiation of cellular features in NPY-stained sections is less distinct, yielding a reduced contrast, though the sympathetic neurons remain positive for both markers.

Sympathetic neurons are positive for TH (Figure 4C). Similarly to the H&E staining scenario (Figure 3), sympathetic neurons appear optically free in both optical and TPM images and can be distinguished from Schwann cells that exhibit indistinct cellular boundaries in the TPM imagery.

Images from TPM tissue sections (Figure 5) reveal sympathetic neurons as a network of elongated, spindle-shaped cell bodies with prominently defined axons and less distinct dendritic branches, characterized by an abundant cytoplasm that consists of microtubules or cytoskeletal structures, alongside a prominent nucleus often located at the narrow periphery of the cell.

Figure 5A,C are sympathetic neurons from PD, Hoehn and Yahr 2, without dysautonomia, and Figure 5B,D are from PD, Hoehn and Yahr 2, with dysautonomia. It is noteworthy that the images of the dysautonomic ganglia, which exhibit clinical manifestations, are microscopically indistinguishable from those of the non-clinically-manifested ganglia observed in PD cases without dysautonomia. This observation implies that the microarchitecture of sympathetic neurons in PD may undergo alterations subsequent to the clinical onset of dysautonomia.

**Figure 6:**
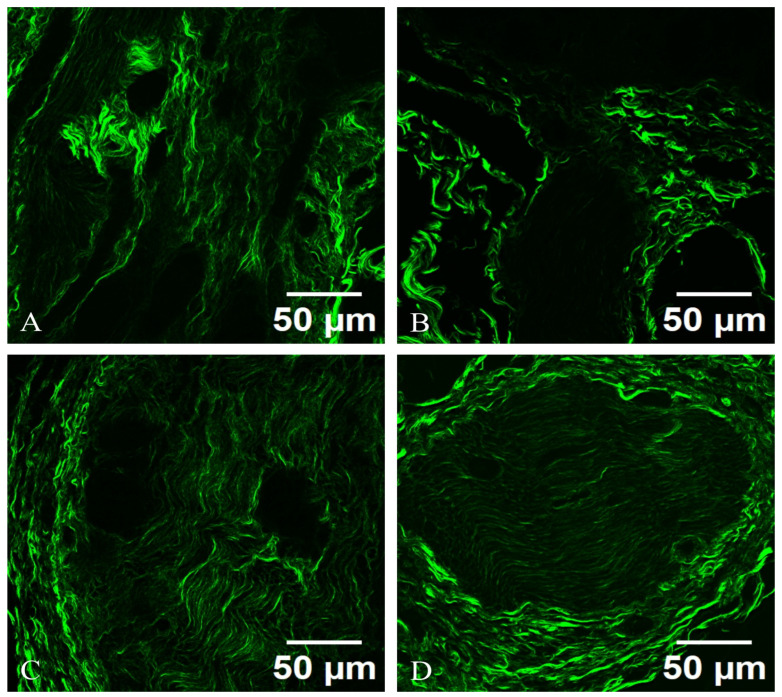
SHG images of sympathetic neurons from celiac ganglion. (**A**,**C**) depict sympathetic neurons from patients with PD, Hoehn and Yahr 4, who do not exhibit dysautonomia, while (**B**,**D**) illustrate sympathetic neurons from patients with PD, Hoehn and Yahr 4, with dysautonomia. SHG signals are displayed in green, showing dense packing of collagen fibers and collagen fibrils, meaning membrane integrity. Importantly, the microscopy images of the ganglia associated with dysautonomia, which clinically manifests, are indistinguishable from those of the non-clinically-manifested ganglia observed in patients with PD without dysautonomia.

Axons are brightly illuminated due to their non-centrosymmetric arrangements, with distinguishable myelinated segments exhibiting a characteristic alternating pattern of SHG intensity, correlating with myelin presence. Surrounding the neurons, a dense network of collagen fibers is visible, indicating the vascular and connective tissue architecture that supports the neuronal environment. The appearance of sympathetic neurons in the TPM images, as light structures with very strong fluorescence, is comparable to their appearance in reflectance confocal microscopy. The sympathetic neurons located in celiac ganglia are visible in both TPM (Figure 5) and SHG (Figure 6).

Under SHG microscopy, a sympathetic neuron from an autonomic ganglion exhibits distinct structural characteristics. The neuron presents a well-defined cell body (soma) with a prominent nucleus and nucleolus, surrounded by processes that extend into dendritic branches. The SHG signal highlights the dense packing of collagen fibers and the extracellular matrix surrounding the neuron, providing insight into the tissue architecture. The axonal projections can be visualized, revealing their interactions with adjacent neurons and glial cells. The nonlinear optical properties of SHG enable the visualization of subcellular structures, such as the synaptic terminals, which appear as fine, fibrillar extensions indicative of neurotransmitter release sites.

The SHG microscopy image reveals the sympathetic neuron from an autonomic ganglion is prominently framed by a network of collagen fibers, which can be distinctly visualized. The collagen matrix appears as bright, organized bundles that provide structural support and delineate the spatial organization of the neuron’s microenvironment. These collagen fibrils often possess a unique orientation, reflecting the directional nature of the nerve fibers and their interactions with the surrounding connective tissue. The collagen not only serves a structural role but also plays a critical role in cell signaling and communication, influencing neuronal growth, repair, and function. The SHG imaging highlights various collagen types, revealing their intricate arrangements and potential interactions with the sympathetic neuron, emphasizing the importance of the extracellular matrix in maintaining neuronal health and facilitating synaptic connectivity in the autonomic nervous system.

The collagen fibers exhibit a striking vibrant green hue, which indicates dense packing and the structural organization inherent to collagen. The collagen appears as long, thin, and wavy fibers, weaving through the image. The fibers may have varying thicknesses, some being more prominent while others are thinner, reflecting the natural heterogenity of collagen within the tissue.

Also, the collagen fibers exhibit a well-defined alignment pattern, which might indicate their organization in relation to the surrounding cellular structures. In some areas, the fibers could appear densely packed and parallel, creating a network that provides mechanical support; in other areas, they may be more dispersed or interwoven, suggesting versatility in function. Some fibers may show branching or anastomosing patterns, indicating how they connect with each other to form a complex three-dimensional matrix. The cross-linking between fibers emphasizes the stability and resilience provided by collagen in the extracellular matrix. The texture of the collagen can appear smooth and continuous in areas, while in others, it may exhibit a more rugged or coarse appearance, depending on the local concentration and structural arrangement of the fibers. In bright regions, the lucidity could indicate areas of higher collagen density, which might be more mechanically robust, as seen in Figure 6.

There are slight fibrotic changes that correspond with distinct modifications in neuronal morphology and synaptic connections. This suggests that dysautonomia-related neurodegeneration leads to alterations in collagen density, orientation, and structural integrity, but later in evolution of PD. This finding suggests that the microarchitectural changes in sympathetic neurons within the context of PD may occur after the clinical onset of dysautonomia.

**Figure 7:**
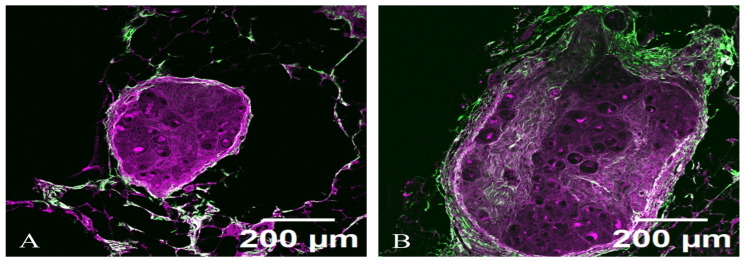
TPM + SHG imaging of celiac ganglia in PD with dysautonomia (**A**) and without dysautonomia (**B**). TPEF signals are shown in magenta–dense cytoplasm with microtubules, dendritic branches, and axons, while SHG signals are displayed in green—dense packing of collagen fibers, collagen fibrils, membrane integrity. Neuronal cell bodies (soma) appear rounded or oval structures, with a dense cytoplasmic outline enhanced by nonlinear optical properties. Dendritic trees extend from the soma, featuring intricate branching and synaptic spines. Axonal projections are visible as elongated, smoother fibers that may reveal pronounced myelinated regions. The soma and myelin sheaths can be highlighted with fluorescent markers for improved visualization.

TPM imaging, with the two contrast mechanisms TPEF and SHG, can provide valuable insights into the structural and functional changes in celiac ganglia in PD, particularly in cases with and without dysautonomia. TPEF allows for the visualization of the soma, dendritic trees, and axonal projections within these ganglia, while SHG imaging can highlight structural components such as collagen and myelin sheaths (Figure 7).

The neuronal cell bodies (soma) appear as rounded or oval structures, possibly displaying a denser cytoplasmic outline due to the nonlinear optical properties being highlighted. The contrasting brightness may enhance features such as the nuclei, which can be evident within the somatic region. Extending from the somas are elaborate dendritic trees, visible as branching processes that capture the complexity of synaptic connections. The branches may appear sharply defined, emphasizing their intricate organization. At certain angles, the processes may cross each other, creating a stunning three-dimensional effect. Axonal projections can be distinguished as elongated fibers, often appearing smoother in texture compared to dendrites. The visualization helps delineate the pathways of neuronal connectivity and may reveal thicker myelinated regions that appear more pronounced due to their SHG signal.

The neuron appears with a central cell body (soma) where the nucleus is located. The soma’s membrane is highlighted with a fluorescent marker, allowing it to stand out against the surrounding tissue. Extending from the cell body are numerous dendritic branches covered in tiny protrusions called dendritic spines, which are responsible for synaptic connections. The detailed structure of these dendrites can be visualized, showing their intricate patterns. The axon of the neuron may also be visible, typically extending away from the soma and displaying a smooth or myelinated appearance, depending on the type of autonomic neuron. The presence of myelin sheaths can be highlighted with specific fluorescent markers.

## 4. Discussion

A significant focus of SHG bioimaging has been on assessing structural changes in the extracellular matrix, particularly in collagen-rich regions, which provide essential insights into the development and progression of various diseases [16]. Due to the widespread presence of collagen in multiple organs, SHG applications have diversified across numerous tissue types, including sympathetic ganglia, human thyroid, skin, pancreas, and lung tissues. The combination of TPEF and SHG has led to various studies showcasing their joint use to unveil intricate tissue characteristics, generating images that highlight vital histological features to assist pathologists in making accurate diagnoses [17].

TPM has opened avenues for research and clinical applications that range from basic studies to diagnostic evaluations. The flexibility of NLO techniques is attributed to their ability to examine fixed, ex vivo, and in vivo tissues [18]. Although one might initially consider that employing NLO techniques on fixed tissues overlaps with traditional bright-field microscopy of labeled samples—which tend to be more cost-effective and accessible—it becomes clear that NLO techniques provide similar insights while offering additional information that enhances conventional histological assessments. Images from NLO microscopy of unlabeled tissues consistently display key features found in traditional histopathology, although it is important to recognize that these features may appear quite different from those observed in bright-field images of stained tissues, such as those prepared with H&E or through IHC staining [19].

The key benefits of using SHG microscopy for investigating celiac ganglia include its ability to image native biological structures without labeling, its high resolution and contrast, and its ability to penetrate deeper into tissues due to its nonlinear optical interactions [20]. SHG offers a powerful platform for studying collagen structure in biological tissues. Its ability to reveal subtle changes associated with diseases makes it a useful tool in biomedical research [21].

TPM was utilized to evaluate sympathetic ganglion tissue sections for features typical of dysautonomia, including sympathetic neurons and Schwann cells and fibroblasts, and the connective tissue capsule, which are the collagen tissue sheaths that surround the components of the nervous structures [22].

SHG microscopy images provide invaluable information regarding the fine structure of sympathetic neurons and their microenvironment. The clarity and detail of the visualization allow for a thorough examination of neuronal integrity and associated connective tissues, contributing insights into both normal physiology and potential pathologies affecting the autonomic nervous system [23]. It is essential to distinguish dysautonomia even before the clinical symptoms, as it can announce future severe neurodegeneration [24].

In light of these findings, a comparative analysis of SHG and TPEF imaging modalities is instrumental in elucidating the specific pathological changes in sympathetic ganglia associated with dysautonomia in PD. Future studies could standardize quantitative metrics for collagen fiber architecture, such as fiber thickness, density, and orientation, alongside neuronal density and morphology parameters. Advanced algorithms and machine learning techniques could facilitate the extraction and analysis of these parameters from SHG and TPEF datasets, potentially leading to the development of predictive models that correlate imaging findings with clinical outcomes. This combined approach would enable more nuanced insights into the timing and nature of pathological changes, allowing researchers to assess the progression of neurodegeneration and its impact on autonomic function.

Longitudinal studies utilizing these imaging techniques could help identify early biomarkers for dysautonomia. Moreover, the utilization of these advanced imaging techniques will provide a critical bridge between cellular-level changes in the nervous system and the clinical manifestations of autonomic dysfunction.

There are many advantages in using TPEF and SHG microscopy. TPEF offers high-resolution imaging and can penetrate deeper into biological tissues compared to conventional fluorescence microscopy due to the use of near-infrared excitation light and is particularly valuable for studying dynamic processes in live cells and tissues, enabling real-time observation of cellular activities and interactions. This technique allows the labeling of specific proteins with fluorescent tags, making it possible to visualize for longer observation times of living tissues.

SHG allows for high-resolution visualization of collagen and other non-centrosymmetric structures without the need for exogenous labels, preserving the native state of biological tissues. The inherent nonlinear optical properties of SHG provide high contrast images that can reveal subtle structural changes, especially in collagen-rich tissues. SHG offers unique insights into the microarchitecture of tissues, providing important information about extracellular matrix organization relevant to disease pathology.

Both TPEF and SHG microscopy provide valuable insights into tissue structure and function, each with distinct disadvantages and limitations. TPEF requires the use of fluorescent labels, which can introduce complexities such as potential artifacts, background noise, and the impact of fluorescence on cell viability. SHG lacks the ability to provide information about specific cellular markers or molecular interactions without complementary tools, such as TPEF.

Despite its advantages, TPM is not yet widespread. This approach also faces challenges; for example, acquiring freshly excised or fixed unlabeled tissues can be difficult, as minimal tissue fragments are typically extracted during medical procedures to minimize patient trauma and scarring. Furthermore, these fragments are generally fixed and stained for use in conventional histopathology diagnostics.

In cases of PD, both with and without dysautonomia, TPEF and SHG reveal a remarkably similar microscopic appearance of celiac ganglia.

In the context of PD, the real-world applications of imaging techniques like TPEF and SHG microscopy can be particularly valuable for understanding the underlying pathophysiology of these symptoms. The ability to visualize microscopic changes in neuronal structure and the extracellular matrix can help identify early biomarkers for dysautonomia. Advanced data analysis techniques, including machine learning, can be applied to imaging datasets obtained from TPEF and SHG to develop predictive models. Such models could correlate specific structural changes in sympathetic neurons and collagen architecture with clinical outcomes, allowing for more personalized patient management strategies.

The use of TPEF and SHG in sympathetic ganglia tissue can provide detailed insights into the morphological characteristics of sympathetic neurons and their microenvironments. This can aid in identifying early pathological changes that may be indicative of neurodegeneration before overt clinical symptoms manifest. Both imaging techniques enable researchers to monitor changes in neuronal morphology and extracellular matrix structures over time, thus, providing insights into how autonomic dysfunction progresses in PD patients. This can help correlate clinical symptoms with structural changes observed in the tissues.

Their real-world applicability spans both clinical diagnostics and cutting-edge research, advancing knowledge in a field that continues to evolve. In research settings, TPEF and SHG microscopy can be invaluable for investigating the underlying mechanisms of neurodegeneration in PD.

Sympathetic neurons in TPM images, both TPEF and SHG, demonstrate a consistent morphology across both groups of PD. Sympathetic neurons appear as elongated cell bodies and processes (axons and dendrites), maintaining their spindle-shaped structure. TPM signals from somatic regions indicate ordered microtubule arrangements or cytoskeletal elements, while dendritic branches intertwine with surrounding structures, although they are less pronounced compared to the axonal extensions.

The axons are prominent in TPM imaging, displaying enhanced brightness due to their non-centrosymmetric arrangement, including neurofilaments or myelin sheaths. Myelinated segments show a characteristic pattern of alternating TPM intensity, correlating with myelin presence. Additionally, the interstitial space exhibits a network of collagen fibers that supports neuronal structures.

In PD with dysautonomia, collagen architecture within the autonomic ganglia did not show increased fibrosis and abnormal remodeling reflected in altered SHG signals. This indicates there are no changes in collagen density, orientation, and structural integrity, resulting from dysautonomia-related neurodegeneration.

While changes in neuronal processes might suggest underlying issues—such as inflammatory responses—the overall microscopic appearance remains strikingly similar between PD with and without dysautonomia. This indicates that although dysautonomia manifests clinically, the fundamental structural integrity at the cellular level may not be drastically different between the two groups. Nonetheless, anomalies in TPM imaging patterns can still hint at pathological changes and neurodegeneration, underlying the importance of using advanced microscopy techniques to investigate these conditions.

Demonstrating that dysautonomia is a functional disorder rather than a structural one is important for several reasons. If dysautonomia is recognized as a functional disorder, treatment approaches can be tailored accordingly. Functional disorders often respond better to therapies that address physiological dysregulation rather than those aimed at structural abnormalities. Establishing that dysautonomia is functional helps refine diagnostic processes. It shifts the focus from identifying structural changes (which are absent) to assessing autonomic functionality through established measures. This can improve diagnostic accuracy and help differentiate dysautonomia from other conditions that may present with similar symptoms. Functional disorders often have different prognoses compared to structural disorders. By framing dysautonomia as functional, clinicians can better inform patients about their condition, likely progression, and response to treatment, which is crucial for patient management and expectations. Recognizing the functional nature of dysautonomia can foster an interdisciplinary approach to treatment, integrating insights from neurology, psychiatry, rehabilitation, and other fields. This collaboration can enhance patient care by addressing the multifaceted aspects of the disorder.

The interplay between IHC and TPM lies in their synergistic application. IHC is used to identify specific proteins of interest within cells, while TPM provides detailed spatial and structural tissue architecture.

The celiac ganglia (PD with dysautonomia and without dysautonomia) exhibit strong positivity for NF-H, NPY, and TH via IHC, coupled with TPM showing normal structural integrity without alterations in collagen, and it suggests that the sympathetic neurons within the ganglia are maintaining their functional capabilities and morphological characteristics. The presence of NF-H indicates robust neuronal architecture, while NPY and TH positivity confirms the functionality of these neurons in catecholamine synthesis and neuropeptide signaling, both critical for autonomic regulation.

Furthermore, the absence of collagen alterations reveals that the extracellular matrix within the ganglia remains intact and provide necessary support for neuronal function, indicating that the connective tissue architecture is adequately supporting nerve homeostasis.

Lack of differences in collagen between these two groups also suggests that dysautonomia in PD is not solely dependent on structural changes in the autonomic nervous system. Specific alterations in collagen, such as increased collagen deposition, lead to a pathological stiffening of the extracellular matrix, potentially impeding the propagation of signals between neurons and altering the mechanosensitivity of the surrounding tissue. Excessive collagen can compress neuronal bodies or disrupt normal cellular interactions, thereby impairing neurotransmitter release and affecting autonomic signaling. Changes in collagen fiber orientation also affect the directional properties of electrical signals. No significant differences are observed in collagen structure between PD with and without dysautonomia, regardless of the PD stage. The autonomic dysfunction is primarily the result of neurotoxic processes where toxic protein aggregates, such as alpha-synuclein, disrupt synaptic transmission but do not alter the structural architecture.

TPEF and SHG imaging of the celiac ganglion visualize changes in collagen structures and alterations in cell signaling pathways without requiring traditional histological staining. This technique provides evidence of functional changes at a cellular level, reinforcing the notion that dysautonomia may be a result of autonomic dysfunction rather than structural damage. This adds a level of specificity and nuances in understanding the neurophysiological mechanisms involved.

## 5. Conclusions

The study highlights the efficacy of TPM, more specifically TPEF and SHG in investigating the micro-structure of celiac ganglia, particularly in the context of dysautonomia associated with PD. Both techniques provide high-resolution imaging capabilities, allowing for detailed visualization of sympathetic neurons and collagen architecture without the need for labeling, thus, overcoming several limitations of classic histopathology. It is significant to note that the microscopic features of dysautonomic ganglia displaying clinical symptoms are indistinguishable from those of non-clinically affected ganglia found in PD patients without dysautonomia. This suggests that the microstructural characteristics of sympathetic neurons in PD experience changes subsequent to the clinical manifestation of dysautonomia. The results indicate that this combined imaging method could facilitate early diagnosis of neurodegeneration and dysautonomia in PD patients, paving the way for potential new studies with therapeutic interventions aimed at improving quality of life. The application of imaging techniques such as TPM represents a connection between neurobiological research and clinical diagnosis, especially within the complex interplay between neurodegenerative disorders and autonomic dysfunctions.

## Figures and Tables

**Table 1:** Participants in cross-sectional study.

Participants	70
PD with Dysautonomia	35
PD without Dysautonomia	35
Women age 45–80	40
Men age 45–80	30
PD stage 2 Hoehn&Yahr, from which:	20
PD stage 2 Hoehn and Yahr with dysautonomia	10
PD stage 2 Hoehn and Yahr without dysautonomia	10
PD stage 3 Hoehn&Yahr from which:	31
PD stage 3 Hoehn and Yahr with dysautonomia	15
PD stage 3 Hoehn and Yahr without dysautonomia	16
PD stage 4 Hoehn&Yahr from which:	19
PD stage 4 Hoehn and Yahr with dysautonomia	10
PD stage 4 Hoehn and Yahr without dysautonomia	9

**Table 2:** Descriptive statistics of Hoehn and Yahr stages.

Descriptive Statistics					
	*N*	Minimum	Maximum	Mean	Std. Deviation
PD Hoehn Yahr 2	20	9	11	10.00	1.414
PD Hoehn Yahr 3	31	14	17	15.50	2.121
PD Hoehn Yahr 4	19	9	10	9.50	0.707
Valid *N* (total)	70				

**Table 3:** Bayesian Estimates of Hoehn and Yahr 2 and Hoehn and Yahr 3, with and without dysautonomia.

Bayesian Estimates of Group Means ^a^					
Dependent Variables	Posterior			95% Credible Interval	
	Mode	Mean	Variance	Lower Bound	Upper Bound
PD Hoehn Yahr 2 with dysautonomia	10.05	10.05	0.085	9.48	10.63
PD Hoehn Yahr 3 with dysautonomia	15.42	15.42	0.085	14.85	15.99
PD Hoehn Yahr 2 without dysautonomia	10.05	4.353	0.09	9	11
PD Hoehn Yahr 3 without dysautonomia	15.42	6.529	0.09	14	17

^a^ Posterior distribution was estimated based on the Bayesian Central Limit Theorem.

**Table 4:** Posterior Distribution Characterization for Poisson Inference.

Posterior Distribution Characterization for Poisson Inference ^a^					
	Mode	Mean	Var.	95% Credible Interval	
				Lower Bound	Upper Bound
PD Hoehn Yahr 2	5.25	5.50	1.375	3.45	8.03
PD Hoehn Yahr 3	8.00	8.25	2.063	5.68	11.29
PD Hoehn Yahr 4	5.00	5.25	1.313	3.25	7.72

^a^ Prior for Poisson Rate/Intensity: Gamma (2, 2).

**Table 5:** Comparison of analyzed parameters according to the existence of dysautonomia.

**Dysautonomia/** **Membrane Integrity**	**Mean ± SD**	**Median (IQR)**	**Mean Rank**	*p* *
Absent	97.23 ± 1.35	97 (96–98)	37.49	0.403
Present	96.89 ± 1.56	97 (96–98)	33.51	
**Dysautonomia/** **Microtubules**	**Mean ± SD**	**Median (IQR)**	**Mean Rank**	*p* *
Absent	95.6 ± 1.95	96 (94–97)	31.71	0.114
Present	96.4 ± 1.66	96 (95–98)	39.29	
**Dysautonomia/** **Synaptic Terminals**	**Mean ± SD**	**Median (IQR)**	**Mean Rank**	*p* *
Absent	95.4 ± 1.89	95 (94–97)	32.73	0.246
Present	95.94 ± 1.6	96 (95–98)	38.27	
**Dysautonomia/** **Prominently Defined Axons**	**Mean ± SD**	**Median (IQR)**	**Mean Rank**	*p* *
Absent	95.83 ± 2.22	96 (94–98)	37.06	0.516
Present	95.57 ± 1.91	95 (94–97)	33.94	
**Dysautonomia/** **Distinct Dendritic Branches**	**Mean ± SD**	**Median (IQR)**	**Mean Rank**	*p* *
Absent	95.51 ± 1.99	96 (94–97)	36.84	0.574
Present	95.31 ± 1.95	95 (94–97)	34.16	
**Dysautonomia/** **Prominent Nucleus**	**Mean ± SD**	**Median (IQR)**	**Mean Rank**	*p* *
Absent	95.2 ± 1.98	95 (94–97)	31.79	0.120
Present	95.91 ± 1.72	95 (95–98)	39.21	
**Dysautonomia/** **Dense Packing-Collagen**	**Mean ± SD**	**Median (IQR)**	**Mean Rank**	*p* *
Absent	95.94 ± 1.91	96 (94–98)	35.44	0.981
Present	96.03 ± 1.72	96 (95–98)	35.56	
**Dysautonomia/** **Collagen Fibrils**	**Mean ± SD**	**Median (IQR)**	**Mean Rank**	*p* *
Absent	95.77 ± 1.76	96 (94–98)	33.63	0.431
Present	96.06 ± 1.71	96 (95–98)	37.37	
**Dysautonomia/** **Myelin Sheaths**	**Mean ± SD**	**Median (IQR)**	**Mean Rank**	*p* *
Absent	95.8 ± 1.90	96 (94–98)	36.27	0.747
Present	95.63 ± 1.64	95 (95–97)	34.73	
**Dysautonomia/** **Cytoplasm**	**Mean ± SD**	**Median (IQR)**	**Mean Rank**	*p* *
Absent	95.63 ± 1.71	95 (94–97)	34.79	0.763
Present	95.71 ± 1.79	95 (95–98)	36.21	

* Mann–Whitney U Test.

## Data Availability

The original contributions presented in this study are included in the article. Further inquiries can be directed to the corresponding authors.

## Abbreviations

The following abbreviations are used in this manuscript:

PD	Parkinson’s Disease
TPM	Two-photon microscopy
TPEF	Two-photon excited fluorescence
NLO	Nonlinear optical microscopy
SHG	Second harmonic generation microscopy
H&E	Hematoxylin and eosin
IHC	Immunohistochemistry
ROI	Region of interest
